# Editorial: Nanoparticle-Mediated Signaling Rewiring and Reprogramming of Immune Responses

**DOI:** 10.3389/fimmu.2022.927733

**Published:** 2022-05-12

**Authors:** Francisco J. Quintana, David Pozo

**Affiliations:** ^1^Ann Romney Center for Neurologic Diseases, Brigham and Women’s Hospital, Harvard Medical School, Boston, MA, United States; ^2^Broad Institute of Massachusetts Institute of Technology (MIT) and Harvard, Cambridge, MA, United States; ^3^Department of Medical Biochemistry, Molecular Biology and Immunology, The University of Seville School of Medicine, Seville, Spain; ^4^CABIMER-Andalusian Center for Molecular Biology and Regenerative Medicine, Seville-University Pablo de Olavide (UPO)-University of Seville-Consejo Superior de Investigaciones Científicas (CSIC), Seville, Spain

**Keywords:** cell signalling, autoimmunity, inflammation, reprogramming, personalized medicine, immunotherapy, nanoparticles, immunoregulation

Recent advances on the molecular mechanisms that control immune cells are at the core of the development of better immunomodulatory therapies. For example, these mechanisms may enable T cell manipulation interventions to treat and prevent autoimmune disease by rewiring T cells towards an anti-inflammatory phenotype. Indeed, it has been recently reported that metabolic sensing in immune cells is coupled to signal transduction pathways that control cell fate. These phenomena have been linked to the onset and/or progression of several diseases such as multiple sclerosis, rheumatoid arthritis, diabetes or Alzheimer’s disease, and also exploited therapeutically to develop cancer immunotherapies (1–4). Unifying our understanding of the molecular pathways that control the immune response might identify novel efficacious interventions to reprogram immune cells for therapeutic purposes in diseases associated with immune dysregulation. However, two important challenges limit the clinical translation of our current knowledge on the regulation of the immune response by small molecules which could provide the basis for novel immunotherapeutic drugs. First, the absence of mechanisms to control the specificity of these reprogramming approaches, resulting in off-target effects. Second, limitations associated to the short half-life and/or low bioavailability of immune-modulatory small molecules.

Nanomaterial-based approaches offer a platform for novel immunotherapeutic approaches. In this sense, nanoparticle (NP) delivery systems are gaining momentum because they allow the development of precision-based medicines for the reprogramming and dynamic rewiring of signalling pathways in immune cells. Even without a well-defined cell-targeting strategy, several studies have shown the feasibility of reprogramming inflammatory immune cells after i.v administration of different nanoparticles based only on their physical properties (5–7). For example, it is possible to differentially reprogram immune cells to favor their homing to injured locations to promote neuroprotection or to reduce their recruitment to inflamed areas and limit immunopathology. Immune-reprogramming NPs have been shown to redirect suppressive macrophages to act as anti-tumor effector cells, and without the described side-effects linked to checkpoint inhibitors. Indeed, several clinical trials are underway to bring immune-reprogramming NPs to patients and there is a critical mass of proof-of-concept studies on nanoparticles tailored with different biological and chemical strategies to modify the response of immune cells (8, 9). Also, NP reprogramming of macrophages and T-cell mediated responses is having a major impact on the development of new methods for cancer immunotherapy (10, 11). In this Research Topic, we have gathered articles covering novel and significant aspects about the connection between NPs and functional immune responses, providing a series of updated and insightful views of the potential mechanisms involved. More specifically, we have arranged this special issue into three broad subjects, as follows: (A) NP-based reprogramming to promote infection resolution. (B) NP-based reprogramming in cancer immunotherapy; (C) NP-based reprogramming of immune memory.

## NP-Based Reprogramming to Promote Infection Resolution

Beyond the bactericidal or bacteriostatic effects that were described in the early days of nanomedicine, an interesting field of research is related to the long-term effect of NP exposure on trained innate immunity (12, 13). Swartzwelter et al. report how gold nanoparticles (AuNPs) loaded with different microbial molecules differentially reprogram macrophage responses which downregulate or exacerbate innate immune memory. Although the molecular mechanisms underlying how AuNPs modulate innate memory are unknown, it appears to be defined by the pathogenic agent and the individual’s immune history, independently of the primary response elicited by AuNPs. An often understudied issue is the linking of initiation, progression and resolution of inflammation with the effect of NPs on functional responses by macrophages. Sun et al. describe a tryptophan-containing hexapeptide-coated AuNP with opposite immunomodulatory activities in resting and TLR-stimulated macrophages, targeting NF-κB or IRAK downstream signalling, respectively. Korshoj et al. provide an insightful review on how to tackle chronic infections in the central nervous system by modulating macrophage/microglia polarization using different NP platforms. Finally, Ernst and Puntes comment on the role of oxidative metabolism in macrophage function and the potential of regulatory interventions trough cerium oxide NPs. Interestingly, it is worth mentioning that inorganic or metallic core NPs are attracting more attention compared to the reluctance they triggered a few years ago because of their potential toxicity.

## NP-Based Reprogramming in Cancer Immunotherapy

The review by Mulens-Arias et al. provides a comprehensive account of our current understanding on the effects of Iron Oxide Nanoparticles (IONPs) on macrophage reprogramming in the tumour microenvironment, including effects on the interactions between malignant and non-transformed cells. Garland et al. explore the capacity of cytosolic double-stranded DNA to promote antitumor immunity by activating the cytosolic DNA sensor cyclic-GMP-AMP synthase (cGAS) and its downstream effector, stimulator of interferon genes (STING), which drive the production of type I interferons and other inflammatory cytokines. A major bottleneck to activate the cGAS/STING cytosolic pathway with double-stranded DNA is the combination of both deoxyribonuclease activity and endosomal escape, which forced the development of direct STING activators. To overcome the limitation of a non-physiological stimulation of cGAS-STING signaling, Garland et al. identified in a DNA library screening a DNA-based polymeric nanoparticle with enhanced features in terms of stability and functional activation of the cGAS-STING pathway. *In vitro* and *in vivo* experiments demonstrated the feasibility of directly targeting cGAS to reprogram macrophages in tumors, opening new venues for cancer immunotherapy. Alongside, Dey et al. provide further information on the impact of nucleic acid cargo in cationic lipid-based delivery systems aimed to induce macrophage and dendritic cell modulation. Finally, Makhijani and McGaha review the role of exosomes in myeloid immune cells. These nano-sized vesicles of endosomal origin (30-150 nm in diameter) are the smallest type of extracellular vesicles, whose role in cancer immunology and inflammatory/autoimmune diseases has only recently started to be fully appreciated.

## NP-Based Reprogramming in Adaptive Immune Memory

The review by Solé and Santamaría focuses on the reprogramming capabilities of NPs to generate antigen-specific T-regulatory type 1 (Tr1)-like cells. They pay special attention to Tr1-like cell heterogeneity and its potential molecular characterization. By using dendritic mesoporous silica nanoparticles, An et al. report the synthesis and characterization of a peptide-based delivery platform as a vaccination strategy towards foot-and-mouth disease virus. Also in the field of modified platforms for new vaccination strategies, Feola et al. exploit oncolytic viruses as *in situ* cancer vaccines. In this work, they developed an alternative approach to generate oncolytic adenovirus functionalised with tumour antigens in order to obtain sustained T cell responses while avoiding non-scalable procedures.

## Author Contributions

Authors listed have made a direct and intellectual contribution to the editorial and approved for publication.

## Funding

DP is supported by the Regional Ministry of Transformación Económica, Industria, Conocimiento y Universidades, PAID2020, CTS677 to DP) and the Spanish Government Ministry of Science and Innovation of Spain [RTI2018-098432-B-I00, co-funded by the European Regional Development Fund (ERDF), a way to build Europe]. FQ is supported by the National Institutes of Health, the Progressive Multiple Sclerosis Alliance and the National Multiple Sclerosis Society.

## Conflict of Interest

The authors declare that the research was conducted in the absence of any commercial or financial relationships that could be construed as a potential conflict of interest.

## Publisher’s Note

All claims expressed in this article are solely those of the authors and do not necessarily represent those of their affiliated organizations, or those of the publisher, the editors and the reviewers. Any product that may be evaluated in this article, or claim that may be made by its manufacturer, is not guaranteed or endorsed by the publisher.
